# Risk of Cardiovascular Disease According to the Precedence Relationship Between Hypertension and Diabetes Mellitus

**DOI:** 10.3390/healthcare13070796

**Published:** 2025-04-02

**Authors:** Junhee Park, Kyungdo Han, Kyuna Lee, Yoosoo Chang, Dong Wook Shin

**Affiliations:** 1 Center for Health Promotion, Samsung Medical Center, Sungkyunkwan University School of Medicine, Seoul 06351, Republic of Korea; junhee.park26@gmail.com; 2 Department of Statistics and Actuarial Science, Soongsil University, Seoul 06978, Republic of Korea; rbskg8274@naver.com; 3 Center for Cohort Studies, Total Healthcare Center, Kangbuk Samsung Hospital, Seoul 04514, Republic of Korea; yoosoo.chang@gmail.com; 4 Department of Occupational and Environmental Medicine, Kangbuk Samsung Hospital, Sungkyunkwan University School of Medicine, Seoul 03181, Republic of Korea; 5 Department of Family Medicine & Supportive Care Center, Samsung Medical Center, Sungkyunkwan University of Medicine, Seoul 06351, Republic of Korea; 6 Department of Digital Health, Samsung Advanced Institute for Health Science & Technology, Sungkyunkwan University, Seoul 06355, Republic of Korea

**Keywords:** cardiovascular, diabetes, hypertension, myocardial infarction, stroke

## Abstract

**Background/Objectives:** Cardiovascular disease (CVD) risk may be based on the sequence of hypertension (HTN) and diabetes mellitus (DM) occurrence since the pathophysiological mechanisms might not be the same. The present study examined the risk of CVD according to the precedent relationship between HTN and DM. **Methods:** Participants with both HTN and DM in a national health screening program in 2015–2016 were divided into two groups based on the order of HTN and DM occurrence: ‘HTN → DM’ and ‘DM → HTN’. The primary outcomes were newly diagnosed myocardial infarction (MI) and ischemic stroke based on the International Classification of Diseases, 10th revision code. **Results:** Among 914,338 participants, there were 28,368 MI events and 35,632 ischemic stroke events during the follow-up period. The DM → HTN group showed a higher risk of MI (adjusted hazard ratio [aHR]: 1.13 [95% CI: 1.10–1.15]) and ischemic stroke (aHR: 1.06 [95% CI: 1.04–1.09]) than the HTN → DM group. The increased risk of MI in the DM → HTN group was more prominent in females than in males and in those without dyslipidemia than in those with dyslipidemia. A higher risk of MI and ischemic stroke in the DM-HTN group was found in patients with chronic kidney disease (CKD) than in patients without CKD. **Conclusions:** MI and ischemic stroke were more frequent in patients in the DM → HTN group than in those of the HTN → DM group. When approaching HTN and DM clinically and epidemiologically, two phenotypes based on the order of occurrence should be considered. Given the generalization limitations of Asian patients, who develop DM at an early age compared to other groups, future studies are needed to reveal the underlying mechanism in the precedence relationship between HTN and DM.

## 1. Introduction

Hypertension (HTN) and diabetes mellitus (DM) are important risk factors for cardiovascular disease (CVD) and are prevalent comorbidities [1]. These conditions have common lifestyle factors and share pathophysiologic pathways such as the renin–angiotensin–aldosterone system (RAAS), inflammation, oxidative stress, and insulin resistance [2]. For example, the local RAAS stimulates vascular inflammation and oxidative stress, which leads to endothelial dysfunction and vascular injury. Increased RAAS activities may cause insulin resistance, which triggers increased production of reactive oxygen species (ROS) and induces free fatty acids, resulting in endothelial dysfunction and atherogenesis [2]. These pathways lead to increased levels of blood pressure and plasma glucose by changing hemodynamic and glycolipid metabolism and interacting with one another, possibly leading to the creation of a vicious cycle. HTN and DM may develop sequentially and often coexist [3]. HTN often precedes DM [4,5,6]. In the Atherosclerosis Risk in Communities (ARIC) Study, the development of type 2 DM (T2DM) was approximately 2.5 times higher in hypertensive than in normotensive patients [7]. Also, individuals with DM showed a two to four times higher risk of HTN than normal patients [8,9]. In Korea, 27.7% of HTN patients aged 20 years or older have been reported to also have DM [10]; 58.6% of patients aged 30 years or older with DM were reported to also have HTN [11].

The co-existence of HTN and DM might correspond to two phenotypes characterized by different temporal trajectories [6]. The sequence of DM and HTN acquisition varies among patients; some patients develop HTN first (HTN → DM), while others develop DM first (DM → HTN). Most epidemiological studies treat HTN and DM as separate independent variables regardless of their order of acquisition, but the pathophysiological mechanisms might not be the same between those who develop HTN first and those who develop DM first.

In addition, chronic kidney disease (CKD) and dyslipidemia, which are closely related to HTN and DM, are strong correlates of CVD occurrence in patients with HTN and DM [12]. However, relevant research on this complex relationship is lacking.

In this regard, we hypothesized that CVD risk might differ between these two groups based on the sequence of HTN and DM occurrence since the pathophysiological mechanisms might not be the same [6]. Thus, in this large cohort study, we tested the hypothesis that CVD risk varies depending on the order of occurrence of HTN and DM with consideration of stratified analysis.

## 2. Materials and Methods

### 2.1. Study Design and Population

This was a population-based nationwide retrospective cohort study using the NHIS database. Among those who underwent health examinations during 2015–2016, those who were ≥20 years old and had both DM and HTN were included in this study (*n* = 1,029,656). DM and HTN statuses were determined by the prescriptions of respective medications under the relevant ICD 10 codes (DM, E11–E14; HTN, I10–I13 and I15) [13]. Patients diagnosed with MI (*n* = 42,440) or ischemic stroke (*n* = 49,734) and those with missing data (*n* = 23,144) were excluded. Therefore, data from 914,338 individuals were included in the analyses.

**Figure 1:**
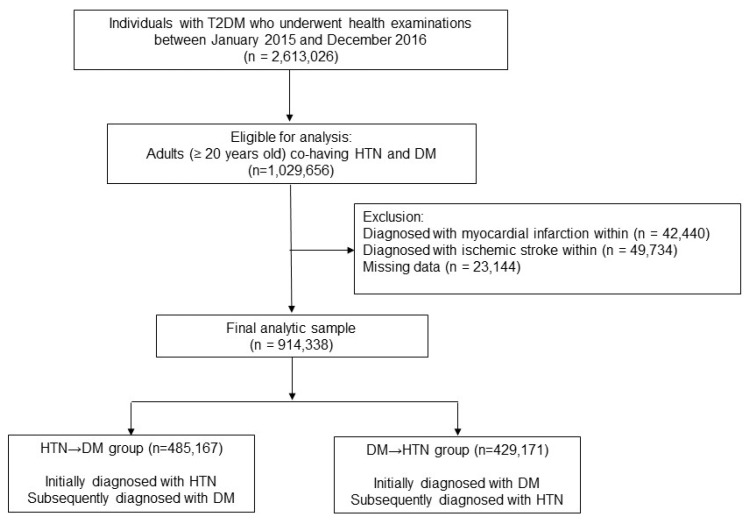
Flowchart of the study.

We divided the patients with co-existing DM and HTN into two groups based on the order in which the individuals contracted the diseases: the ‘HTN → DM’ group and the ‘DM → HTN’ group. The sequence of disease acquisition was determined by prescription history. The ‘HTN → DM’ group was defined as those initially diagnosed with HTN and subsequently diagnosed with DM; the ‘DM → HTN’ group was defined as those diagnosed with DM first (Figure 1). The duration of HTN and DM were defined from initial drug prescriptions to the date of health examinations. The present study complied with the ethical rules for human experimentation described in the Declaration of Helsinki and was approved by the Institutional Review Board (IRB) of Samsung Medical Center (IRB File No. SMC 2022-07-072). Patient consent was waived because of the anonymized structure of the National Health Insurance Service (NHIS) research database used in this study.

### 2.2. Study Outcomes

Newly diagnosed MI or ischemic stroke were the primary endpoints in this study. Based on the ICD-10 codes, cases of newly diagnosed MI (I21 or I22 during hospitalization or recording of these codes at least twice in an outpatient setting) or ischemic stroke (I63 or I64 during hospitalization with claims for brain magnetic resonance imaging or brain computerized tomography) were identified [13]. The cohort followed the events of incident MI and ischemic stroke until the end of the study period (31 December 2020).

### 2.3. Covariates

Household income was dichotomized by the lowest 20th percentile according to the health insurance premium level based on income level under the social health insurance system in Korea. CKD was defined as a glomerular filtration rate <60 mL/min per 1.73 m^2^, as estimated by the Modification of Diet in Renal Disease equation [14]. Dyslipidemia was defined as a history of a claim with an E78 code and receipt of lipid-lowering medications or a total cholesterol level ≥240 mg/dL. Smoking status was classified as a non-smoker, ex-smoker, or current smoker. Alcohol consumption was categorized as none, mild to moderate (<30 g/day), or heavy (≥30 g/day) [15]. Regular physical activity was defined as high-intensity physical activity causing extreme shortness of breath for >20 min/session, ≥3 days/week, and/or moderate-intensity physical activity that causes substantial shortness of breath for >30 min/session, ≥5 days/week [16,17]. Body mass index was calculated as body mass in kilograms divided by the square of height in meters.

### 2.4. Statistical Analysis

Baseline characteristics were analyzed by descriptive statistics. Continuous variables are presented as mean ± standard deviations (SDs) for normally distributed data. Geometric means and 95% CI values are presented for non-normally distributed triglyceride levels. Categorical variables are presented as numbers and percentages. Hazard ratios (HRs) and 95% CI values for MI and ischemic stroke were analyzed using a multivariable Cox proportional hazards model using Schoenfeld residuals. Model 1 was adjusted for age and sex. Model 2 was further adjusted for BMI, income, smoking status, alcohol consumption, physical activity level, and dyslipidemia or CKD. Model 3 was further adjusted for systolic blood pressure and fasting glucose level. Model 4 was further adjusted for DM duration and HTN duration. A one-year lag sensitivity analysis was conducted to examine the potential for surveillance bias (Supplementary Table S1). Stratified analyses by age, sex, dyslipidemia, and CKD were performed to determine the potential interactions between the two groups and the incidence of MI and ischemic stroke. SAS version 9.4 (SAS Institute Inc., Cary, NC, USA) was used for statistical analyses, and a *p*-value < 0.05 was considered statistically significant.

## 3. Results

### 3.1. Characteristics of Study Participants

The DM → HTN group was more likely to be younger, male, less frequently obese, ex- or current smokers, and mild or heavy alcohol consumers than the HTN → DM group. The DM first group showed lower BP in both systolic and diastolic phases but higher fasting glucose levels and more frequent insulin use than the HTN → DM group. The DM → HTN group had a greater prevalence of CKD but a lower prevalence of dyslipidemia and lower lipid profiles than the HTN → DM group (Table 1).

### 3.2. Risk of Study Outcomes

During the follow-up period, a total of 28,368 MI events (13,829 in the HTN → DM group and 14,539 in the DM → HTN group) and 35,632 ischemic stroke events (17,868 in the HTN → DM group and 17,764 in the DM → HTN group) were identified. Individuals in the DM → HTN group had an increased risk of MI (adjusted HR [aHR]: 1.13, [95% CI: 1.10–1.15]) and ischemic stroke (aHR: 1.06, [95% CI: 1.04–1.09]) compared to those in the HTN → DM group (Table 2). Sensitivity analysis with a one-year lag time supports the robustness of these results (Supplementary Table S1).

### 3.3. Stratified Analysis by Age, Sex, Dyslipidemia, and Chronic Kidney Disease

In the age-specific stratification analysis, the increased risk of ischemic stroke in DM → HTN patients was more prominent in those aged 40–64 years than in those ≥ 65 years (aHR: 1.13 [95% CI, 1.09–1.18] vs. 1.04 [1.01–1.06], *p* for interaction = 0.0005). No significant results were found in 20–39-year-olds due to the small number involved in the study. The increased risk of MI in the DM → HTN group was more prominent in females and those without dyslipidemia than in males and those with dyslipidemia. In the stratification analysis by CKD, patients with CKD showed more frequent significant interactions with both MI and ischemic stroke (MI: *p* for interaction = <0.0001, ischemic stroke: *p* for interaction = 0.0003) than those without CKD (Table 3).

## 4. Discussion

In this study, we first assessed the risk of MI and ischemic stroke according to the order of occurrence of HTN and DM. The pathophysiological mechanisms of CVD may differ between patients in the HTN → DM and DM → HTN groups, and the risk of CVD was higher when DM occurred first. The DM → HTN group showed a 13% and 6% higher risk of MI and ischemic stroke, respectively, than the HTN → DM group. The sensitivity analysis confirms the consistency of our findings.

Blood pressure, obesity, and cholesterol levels, which are risk factors for CVD, were expected to be better controlled in the DM → HTN group, probably because of the strict DM guidelines regarding the strict control of blood pressure and lipid levels [18]. However, poor glycemic control and renal function decline caused by insulin resistance, which gradually emerges over time after diagnosis of DM [19], were present in some DM → HTN group patients. One possible inference is that the DM → HTN group and the HTN → DM group have different insulin resistance periods. Insulin resistance, hyperinsulinemia, increased oxidative stress, and subclinical chronic inflammation are the most studied pathophysiological mechanisms between HTN and DM [20]. Insulin resistance, a complex and multifaceted syndrome that can affect blood pressure homeostasis, may be pivotal in the development of diabetes [20].

DM → HTN patients might possess the ‘insulin resistance phenotype’ in a complex cause–effect relationship between HTN and DM [6]. The insulin resistance of endothelial cells from initial DM increases pro-thrombotic and pro-inflammatory factors and ROS, resulting in endothelial cell dysfunction [21]. Persistent hyperglycemia can cause mitochondrial dysfunction, leading to an inflammatory response and cellular damage by increasing ROS [22]. When insulin signaling is impaired, the effects at the cellular level cannot be fully recovered, even though improvements are achieved [23]. Persistent cellular effects may cause cellular changes and damage from the initial onset of insulin resistance. This can lead to a lasting impact on cellular function and an increased risk of atherosclerosis and CVD [23]. Furthermore, RAAS activation and hyperinsulinemia due to insulin resistance can act synergistically in stimulating the mitogen-activated protein kinase (MAPK) [24]. This pathway can induce endothelial dysfunction and promote atherosclerosis.

HTN → DM patients correspond to a mixture of an ‘insulin resistance phenotype’ and a ‘vascular damage phenotype.’ In the short term, newly developed DM might act as an ‘insulin resistance phenotype’; but in the long term, newly developed DM may be a result of extensive organ damage due to HTN. Two distinct entities of subsequent DM to HTN (early and late) previously exhibited different risks of mortality and cardiovascular mortality, and the overall prognosis was primarily HTN-dependent [25]. For example, HTN can increase response to stimulation of the vasoconstrictor, which leads to impaired vasodilation of the skeletal muscle. This results in vasoconstriction, which can contribute to the genesis of vascular structural changes and increase the number of fast-twitch fibers, which may contribute to the development of insulin resistance. Changes in endothelial permeability and decreased peripheral blood flow due to HTN may limit insulin delivery and promote insulin resistance, which impairs glucose uptake. In addition, systemic vascular resistance accompanied by oxidative stress and inflammation activates signaling molecules, such as nuclear factor–kappa B (NF-ĸB), and other mediators of stress-sensitive pathways, such as interleukin-1 and -6 and tumor necrosis factor-alpha (TNF-α), all of which can modify glucose and lipid metabolism, increase insulin resistance, and lead to DM [26]. The physical stress on the arterial wall caused by initial HTN results in the deterioration and acceleration of atherosclerosis, particularly affecting coronary and cerebral vessels [27]. Thus, mechanical stress, rather than insulin resistance, may be the main factor that leads to CVD in HTN → DM patients [28]. Additionally, newly developed DM patients are prone to blood clots due to changes in platelet count and activation, as well as modifications of coagulation and fibrinolytic factors, all of which can magnify the risk of CVD [29].

The difference in outcome occurrence between the two phenotype groups was higher in MI than in stroke, one interpretation of which is that the risk of coronary heart disease is greater in DM than in HTN [30]. This could explain the difference in MI between the DM → HTN group with a longer duration of DM and the HTN → DM group with relatively weaker DM effects. Stroke, however, is more affected by HTN than by DM [31]. In our study design, the HTN → DM group contained those with the ‘insulin resistance phenotype’; this explains the relatively small difference in risk between the groups.

To determine the reason for the higher risk of CVD in the DM → HTN group than the HTN → DM group, whether it is because of the sequence of development of DM or because of the longer progression of DM, we performed exploratory analysis by further adjusting for the duration of HTN and DM (Model 4 in Table 2). While attenuated, significant associations remained. This finding suggests that the duration of HTN and DM at least partially explains the risk difference between the two groups.

The increased risk of ischemic stroke in patients in the DM → HTN group was more pronounced in those who were 40–64 years of age than in those aged 65 and older. Since stroke has a low risk in the young and increases steeply in old age [32], the effect of DM may have been more pronounced in the relatively young age group. However, the findings were not significant in our 20–39-year-olds due to a lack of patients. The increased risk of MI in DM → HTN patients showed a similar trend but was also not significant. This might be due to the cascading risk of MI [33], unlike ischemic stroke, in which the risk increases rapidly in the elderly [32]. The increased risk of MI in the DM → HTN group was more pronounced in women than in men; this may explain the relatively greater effect of insulin resistance on CVD. This result is consistent with the findings of the Atherosclerosis Risk in Communities study that HTN and DM were greater contributors to CVD risk in women compared with men [34]. The reasons for sex differences are not yet clear [35], but these differences may be due to a lack of awareness or treatment of the prevalent risk factors in women. A yet-to-be-identified hormonal or non-hormonal biological variation may also be responsible for the difference [36]. Additionally, men could have a more inherent unexplained risk of CVD, such as nonconventional risk factors like HTN and DM. In the same context, the difference in insulin resistance to CVD was relatively greater in patients without dyslipidemia, which accounted for a large proportion of those with CVD risk because the underlying risk was lower than in those with dyslipidemia [37]. The higher risk of MI or ischemic stroke in the DM-HTN group was more prominent in patients with chronic kidney disease (CKD) than in patients without CKD. The prevalence of CVD is twice as high in patients with CKD and is stated to have a significantly higher CVD risk than DM [38]. CKD can be considered a CVD equivalent [39]. Therefore, in CKD patients, the markedly increased risk of MI and stroke in the DM → HTN group is as predicted.

There are several limitations that should be considered. First, the findings may be subject to selection bias. As the study was limited to participants who underwent health screening examinations, the participants may tend to have healthier lifestyles than the general population. Second, HTN and DM can be initially treated with lifestyle modifications, such as weight reduction, dietary change, or increased physical activity. Since our definitions were based on prescribed medications, it can be difficult to identify the exact timing of diagnosis. In addition, we did not analyze type 1 DM with different pathogeneses. The two different types of DM might produce different outcomes. Third, we could not assess the exact biological mechanisms in the precedence relationship between HTN and DM. Also, the information related to CVD risk regarding insulin resistance, such as homeostasis model assessment for insulin resistance (HOMA-IR) and vascular elasticity, could not be collected. Fourth, there could be unmeasured residual confounding factors such as the exact degree of glycemic control (e.g., glycated hemoglobin). Finally, the study population consisted of Koreans; this limits the generalizability of our findings. Asian patients develop DM at an early age compared to other groups and are characterized by early β-cell dysfunction with insulin resistance [40]. East Asian patients with T2DM have a higher risk of renal complications than Europeans and are likely to develop strokes as cardiovascular complications [40]. Therefore, further studies are required to confirm the risk of CVD according to the precedence relationship between HTN and DM and generalize the findings to other ethnic backgrounds.

## 5. Conclusions

In the present study, we made the first attempt to examine CVD risk based on the precedent relationship between HTN and DM. MI and ischemic stroke outcomes were higher in DM → HTN patients than in HTN → DM patients. Well-structured investigations in the future could unveil the complicated mechanism in the precedence relationship between HTN and DM, including insulin resistance and vascular elasticity.

## Figures and Tables

**Table 1:** Baseline characteristics of the study population.

Variable	HTN → DM Group (*n* = 485,167)	DM → HTN Group (*n* = 429,171)	*p*-Value
Age, years	64.7 ± 10.2	63.7 ± 10.6	<0.001
20–39	3412 (0.7)	5059 (1.2)	<0.001
40–64	237,078 (48.9)	221,362 (51.6)	
≥65	244,677 (50.4)	202,750 (47.2)	
Sex, male	246,137 (50.7)	257,227 (59.9)	<0.001
Income, lowest 20%	104,704 (21.6)	96,091 (22.4)	<0.001
HTN duration, years	10.7 ± 3.4	7.9 ± 4.3	<0.001
<1	3207 (0.7)	26,532 (6.2)	<0.001
<5	39,026 (8.0)	100,352 (23.4)	
<10	124,914 (25.8)	138,713 (32.3)	
≥10	318,020 (65.6)	163,574 (38.1)	
DM duration, years	5.8 ± 3.8	10.5 ± 3.8	<0.001
<1	50,463 (10.4)	6738 (1.57)	<0.001
<5	177,236 (36.5)	46,351 (10.8)	
<10	173,405 (35.7)	100,794 (23.5)	
≥10	84,063 (17.3)	275,288 (64.1)	
Insulin use	35,921 (7.4)	81,803 (19.1)	<0.001
Comorbidity			
Chronic kidney disease	68,709 (14.2)	81,727 (19.0)	<0.001
Dyslipidemia	333,621 (68.8)	284,138 (66.2)	<0.001
Smoking			<0.001
Non-smoker	307,873(63.5)	245,296 (57.2)	
Ex-smoker	102,439 (21.1)	101,852 (23.7)	
Current smoker	74,855 (15.4)	82,023 (19.1)	
Alcohol consumption			<0.001
None	319,346 (65.8)	270,712 (63.1)	
Mild to moderate	130,686 (26.9)	122,841 (28.6)	
Heavy	35,135 (7.2)	35,618 (8.3)	
Physical activity, regular	103,902 (21.4)	96,566 (22.5)	<0.001
Height, cm	160.4 ± 9.3	161.9 ± 9.3	<0.001
Weight, kg	67.5 ± 12.4	66.4 ± 12.0	<0.001
Body mass index, kg/m^2^	26.1 ± 3.6	25.2 ± 3.4	<0.001
Waist circumference, cm	88.1 ± 9.0	86.9 ± 8.7	<0.001
Systolic BP, mmHg	131.7 ± 15.0	130.0 ± 15.1	<0.001
Diastolic BP, mmHg	78.9 ± 9.8	76.9 ± 9.7	<0.001
Laboratory findings			
Glucose, fasting, mg/dL	131.0 ± 37.4	145.3 ± 49.2	<0.001
Total cholesterol, mg/dL	173.7 ± 38.6	170.1 ± 39.1	<0.001
Triglycerides *, mg/dL	134.2 (134.0–134.4)	130.0 (129.8–130.2)	<0.001
HDL-C, mg/dL	50.2 ± 13.7	49.4 ± 13.7	<0.001
LDL-C, mg/dL	93.5 ± 34.6	91.4 ± 34.5	<0.001
eGFR	84.9 ± 48.0	82.5 ± 49.9	<0.001

Values are expressed as mean ± standard deviations or numbers (%). * Values are presented as geometric means (95% confidence interval). Abbreviations: BP, blood pressure; HDL-C, high-density lipoprotein cholesterol; LDL-C, low-density lipoprotein cholesterol; eGFR, estimated glomerular filtration rate.

**Table 2:** Hazard ratios and 95% confidence intervals of study outcomes according to the precedence relationship between hypertension and diabetes mellitus.

	No. (%)	Case No.	Duration, PY	IR, 1000PY	Model 1 aHR (95% CI)	Model 2 aHR (95% CI)	Model 3 aHR (95% CI)	Model 4 aHR (95% CI)
Myocardial infarction								
HTN → DM group	485,167 (53.1)	13,829	2,329,520	5.9	1 (Ref.)	1 (Ref.)	1 (Ref.)	1 (Ref.)
DM → HTN group	429,171 (46.9)	14,539	2,050,611	7.1	1.22 (1.19, 1.25)	1.15 (1.13, 1.18)	1.13 (1.10, 1.15)	1.05 (1.01, 1.09)
Ischemic stroke								
HTN → DM group	485,167 (53.1)	17,868	2,320,096	7.7	1 (Ref.)	1 (Ref.)	1 (Ref.)	1 (Ref.)
DM → HTN group	429,171 (46.9)	17,764	2,042,764	8.7	1.16 (1.14, 1.19)	1.11 (1.08, 1.13)	1.06 (1.04, 1.09)	1.03 (1.00, 1.07)

Abbreviations: DM, diabetes mellitus; HTN, hypertension; PY, person-years; IR, incidence rate; HR, hazard ratio; aHR, adjusted hazard ratio; CI, confidence interval. Model 1: adjusted for age and sex. Model 2: Model 1 plus further adjusted for body mass index, income, smoking, alcohol consumption, regular physical activity, dyslipidemia, and chronic kidney disease. Model 3: Model 2 plus further adjusted for systolic blood pressure and fasting glucose. Model 4: Model 3 plus further adjusted for DM duration and HTN duration.

**Table 3:** Risk of study outcomes according to the precedence relationship between hypertension and diabetes mellitus stratified by age, sex, dyslipidemia, and CKD.

Study Outcomes	Stratification	Study Group	No. (%)	Case No.	Duration, PY	IR, 1000PY	aHR (95% CI)	*p* for Interaction
	Age							
Myocardial infarction	20–39 years	HTN → DM	3412 (40.3)	37	16,642	2.2	1 (Ref.)	0.13
		DM → HTN	5059 (59.7)	50	24,893	2.1	0.88 (0.58, 1.35)	
	40–64 years	HTN → DM	237,078 (51.7)	4637	1,155,025	4.0	1 (Ref.)	
		DM → HTN	221,362 (48.3)	5458	1,074,787	5.1	1.16 (1.11, 1.20)	
	≥65 years	HTN → DM	244,677 (54.7)	9155	1,157,854	7.9	1 (Ref.)	
		DM → HTN	202,750 (45.3)	9031	950,932	9.5	1.11 (1.08, 1.14)	
Ischemic stroke	20–39 years	HTN → DM	3412 (40.3)	25	16,658	1.5	1 (Ref.)	<0.001
		DM → HTN	5059 (59.7)	35	24,929	1.4	0.92 (0.55, 1.53)	
	40–64 years	HTN → DM	237,078 (51.7)	4800	1,154,610	4.2	1 (Ref.)	
		DM → HTN	221,362 (48.3)	5611	1,074,204	5.2	1.13 (1.09, 1.18)	
	≥65 years	HTN → DM	244,677 (54.7)	13,043	1,148,828	11.3	1 (Ref.)	
		DM → HTN	202,750 (45.3)	12,118	943,632	12.8	1.04 (1.01, 1.06)	
	Sex							
Myocardial infarction	Male	HTN → DM	246,137 (48.9)	7107	1,176,497	6.0	1 (Ref.)	<0.01
		DM → HTN	257,227 (51.1)	8478	1,225,585	6.9	1.09 (1.06, 1.13)	
	Female	HTN → DM	239,030 (58.2)	6722	1,153,024	5.8	1 (Ref.)	
		DM → HTN	171,944 (41.8)	6061	825,026	7.3	1.17 (1.13, 1.21)	
Ischemic stroke	Male	HTN → DM	246,137 (48.9)	9227	1,171,473	7.9	1 (Ref.)	0.13
		DM → HTN	257,227 (51.1)	10,472	1,220,565	8.5	1.05 (1.02, 1.08)	
	Female	HTN → DM	239,030 (58.2)	8641	1,148,623	7.5	1 (Ref.)	
		DM → HTN	171,944 (41.8)	7292	822,199	8.9	1.08 (1.05, 1.12)	
	Dyslipidemia							
Myocardial infarction	Dyslipidemia	HTN → DM	151,546 (51.1)	4497	723,837	6.2	1 (Ref.)	<0.05
		DM → HTN	145,033 (48.9)	4864	689,696	7.1	1.08 (1.04, 1.13)	
	No dyslipidemia	HTN → DM	333,621 (54.0)	9332	1,605,683	5.8	1 (Ref.)	
		DM → HTN	284,138 (46.0)	9675	1,360,915	7.1	1.15 (1.11, 1.18)	
Ischemic stroke	Dyslipidemia	HTN → DM	151,546 (51.1)	6341	719,644	8.8	1 (Ref.)	0.15
		DM → HTN	145,033 (48.9)	6572	685,751	9.6	1.04 (1.01, 1.08)	
	No dyslipidemia	HTN → DM	333,621 (54.0)	11,527	1,600,452	7.2	1 (Ref.)	
		DM → HTN	284,138 (46.0)	11,192	1,357,013	8.2	1.08 (1.05, 1.10)	
	CKD							
Myocardial infarction	CKD	HTN → DM	68,709 (45.7)	3240	318,663	10.2	1 (Ref.)	<0.001
		DM → HTN	81,727 (54.3)	4962	373,192	13.3	1.30 (1.24, 1.36)	
	No CKD	HTN → DM	416,458 (54.5)	10,589	2,010,858	5.3	1 (Ref.)	
		DM → HTN	347,444 (45.5)	9577	1,677,420	5.70	1.06 (1.03, 1.09)	
Ischemic stroke	CKD	HTN → DM	68,709 (45.7)	4312	316,264	13.6	1 (Ref.)	<0.001
		DM → HTN	81,727 (54.3)	5739	371,329	15.5	1.13 (1.09, 1.18)	
	No CKD	HTN → DM	416,458 (54.5)	13,556	2,003,832	6.8	1 (Ref.)	
		DM → HTN	347,444 (45.5)	12,025	1,671,435	7.2	1.04 (1.01, 1.06)	

Abbreviations: DM, diabetes mellitus; HTN, hypertension; CKD, chronic kidney disease; PY, person-years; IR, incidence rate; HR, hazard ratio; aHR, adjusted hazard ratio; CI, confidence interval. Adjusted for age, sex, body mass index, income, smoking, alcohol consumption, regular physical activity, dyslipidemia, chronic kidney disease, systolic blood pressure, and fasting glucose.

## Data Availability

The data that support the findings of this study are available from the Korean National Health Insurance Service (KNHIS) and were used under license for the current study (http://nhiss.nhis.or.kr, accessed on 30 July 2023). However, restrictions apply to their availability; the data are not publicly available. Data are available from the authors upon reasonable request and with the permission of the KNHIS.

## Supplementary Materials

The following supporting information can be downloaded at: https://www.mdpi.com/article/10.3390/healthcare13070796/s1, Supplementary Method S1: Data source; Supplementary Table S1: Sensitivity analysis: Hazard ratios and 95% confidence intervals of study outcomes according to the precedence relationship between hypertension and diabetes mellitus with a 1-year lag time. References [41,42] are cited within the Supplementary Materials.
