# Supplementary material for: Risk of Cardiovascular Disease According to the Precedence Relationship Between Hypertension and Diabetes Mellitus
Source: Healthcare (Basel). 2025 Apr 2;13(7):796. doi: 10.3390/healthcare13070796 (PMC11989093; doi:10.3390/healthcare13070796)
Supplement: Supplementary file 1 [file healthcare-13-00796-s001.zip › healthcare-3542591-supplementary.pdf]

Supplementary Online Content

Supplementary materials

Supplementary Method S1. Data source

Supplementary Table S1. Sensitivity analysis: Hazard ratios and 95% confidence intervals of study outcomes according to the precedence relationship between hypertension and diabetes mellitus with a 1-year lag time

This supplementary material has been provided by the authors to give readers additional information about their work.

Supplementary Methods. Data source

The Korean National Health Insurance Service (NHIS) is the Korean government's mandatory universal medical insurer that covers almost the entire population (97%). The 3% in the lowest income bracket are Medicaid beneficiaries. The NHIS provides a biennial national health examination program to all insured beneficiaries aged over 40 years and employees regardless of age. The health examination consists of a self-administered health questionnaire that includes lifestyle information (i.e., smoking status, alcohol consumption, and physical activity), anthropometric measurements (i.e., height, body mass, and blood pressure [BP]), and laboratory test data (i.e., serum glucose, lipid profile, and health indicators). There is a standard measurement protocol for general health screening program [1]. For example, BP should be measure in a quiet environment by auscultation or oscilloscopic automatic sphygmomanometer with a at least 5minutes rest in a seated position prior to the measurement. In addition, the NHIS database contains data regarding demographics and utilization of inpatient and outpatient medical services including International Classification of Disease, 10th revision (ICD-10), diagnosis codes and prescriptions [2].

Reference

1. Shin, D.W.; Cho, J.; Park, J.H.; Cho, B. National General Health Screening Program in Korea: history, current status, and future direction. *Precis. Future Med.* **2022**, *6*, 9–31.
2. Cheol Seong, S.; Kim, Y.Y.; Khang, Y.H.; Heon Park, J.; Kang, H.J.; Lee, H.; Do, C.H.; Song, J.S.; Hyon Bang, J.I.; Ha, S.; et al. Data Resource Profile: The National Health Information Database of the National Health Insurance Service in South Korea. *Int. J. Epidemiol.* **2017**, *46*, 799–800.

Supplementary Table S1. Sensitivity analysis: Hazard ratios and 95% confidence intervals of study outcomes according to the precedence relationship between hypertension and diabetes mellitus with a 1-year lag time

	No. (%)	Case No.	Duration, PY	IR, 1000PY	Model 1 aHR (95% CI)	Model 2 aHR (95% CI)	Model 3 aHR (95% CI)	Model 4 aHR (95% CI)
Myocardial infarction								
HTN→DM group	476,289 (53.1)	11,340	1,836,621	6.2	1 (Ref.)	1 (Ref.)	1 (Ref.)	1 (Ref.)
DM→HTN group	419,843 (46.9)	11,912	1,614,096	7.4	1.23 (1.19, 1.26)	1.16 (1.13, 1.19)	1.13 (1.10, 1.16)	1.05 (1.01, 1.09)
Ischemic stroke								
HTN→DM group	476,289 (53.1)	14,308	1,831,284	7.8	1 (Ref.)	1 (Ref.)	1 (Ref.)	1 (Ref.)
DM→HTN group	419,843 (46.9)	14,144	1,610,138	8.8	1.16 (1.13, 1.18)	1.11 (1.08, 1.13)	1.06 (1.04, 1.09)	1.04 (1.00, 1.07)

Abbreviations: DM, diabetes mellitus; HTN, hypertension; PY, person-years; IR, incidence rate; HR, hazard ratio; aHR, adjusted hazard ratio; CI, confidence interval. Model 1: adjusted for age and sex. Model 2: Model 1 plus further adjusted for body mass index, income, smoking, alcohol consumption, regular physical activity, dyslipidemia, and chronic kidney disease. Model 3: Model 2 plus further adjusted for systolic blood pressure and fasting glucose. Model 4: Model 3 plus further adjusted for DM duration and HTN duration.
